# A 12-week consumer wearable activity tracker-based intervention reduces sedentary behaviour and improves cardiometabolic health in free-living sedentary adults: a randomised controlled trial

**DOI:** 10.1186/s44167-022-00007-z

**Published:** 2022-12-01

**Authors:** Wouter M. A. Franssen, Ine Nieste, Frank Vandereyt, Hans H. C. M. Savelberg, Bert O. Eijnde

**Affiliations:** 1 REVAL-Rehabilitation Research Center, Faculty of Rehabilitation Sciences, Hasselt University, Hasselt, Belgium; 2 SMRC-Sports Medicine Research Center, BIOMED-Biomedical Research Institute, Faculty of Medicine and Life Sciences, Hasselt University, Hasselt, Belgium; 3 Department of Nutrition and Movement Sciences, NUTRIM, School for Nutrition and Translation Research Maastricht, Faculty of Health, Medicine and Life Sciences, Maastricht University, Maastricht, The Netherlands; 4 Department of Cardiology, Virga Jessa Hospital, Heart Centre Hasselt, Hasselt, Belgium

**Keywords:** Activity tracker, Wearables, Sedentary behaviour, Cardiometabolic health, Chronic disease

## Abstract

**Background:**

Reducing sedentary behaviour significantly improves cardiometabolic health and plays an important role in the prevention and management of cardiometabolic diseases. However, limited effective strategies have been proposed to combat the negative effects of sedentary lifestyles. Although consumer wearable activity trackers (CWATs) can effectively improve physical activity, they were only included as part of a multiple behaviour change technique. In addition, it is not known whether these devices are also effective to reduce sedentary behaviour. Therefore, we aim to investigate the efficacy of a single component CWAT-only intervention and the added value of a multicomponent (CWATs + motivational interviewing) behaviour change intervention to reduce sedentary behaviour and increase physical activity within sedentary adults.

**Methods:**

In a three-armed randomised controlled trial, 59 (male/female: 21/38) sedentary adults were randomly allocated to a control group (n = 20), a CWAT-only group (n = 20) or the CWAT + group (CWAT + motivational interviewing; n = 19) for 12 weeks. Physical activity and sedentary behaviour were assessed using the activPAL3™ accelerometer. In addition, anthropometrics, blood pressure, plasma lipids and insulin sensitivity using an oral glucose tolerance test were assessed at baseline and after the 12-week intervention period.

**Results:**

As compared with the control group, the CWAT + group significantly reduced time spent in sedentary behaviour (− 81 min/day, confidence interval [95%]: [− 151, − 12] min/day) and significantly increased step count (+ 3117 [827, 5406] steps/day), standing time (+ 62 [14, 110] min/day), light intensity PA (+ 28 [5, 50] min/day) and moderate-to-vigorous PA (+ 22 [4, 40] min/day). Body fat mass (− 1.67 [− 3.21, − 0.14] kg), percentage body fat (− 1.5 [− 2.9, − 0.1] %), triglyceride concentration (− 0.31 [− 0.62, − 0.01] mmol/l), the 2 h insulin concentration (− 181 [− 409, − 46] pmol/l), the quantitative insulin sensitivity check index (− 0.022 [− 0.043, − 0.008]) and total area under the curve of insulin (− 6464 [− 26837, − 2735] mmol/l min) were significantly reduced in the CWAT + group, compared to the control group. No significant differences within the CWAT-only group were found.

**Conclusion:**

A 12-week multicomponent CWAT-based intervention (CWAT + motivational interviewing) reduces sedentary time, increases physical activity levels and improves various cardiometabolic health variables in sedentary adults, whereas self-monitoring on itself (CWAT-only group) has no beneficial effects on sedentary time.

*Trial registration* The present study was registered (2018) at clinicaltrials.gov as NCT03853018.

**Supplementary Information:**

The online version contains supplementary material available at 10.1186/s44167-022-00007-z.

## Introduction

The metabolic syndrome represents a cluster of cardiometabolic risk factors including insulin resistance, abdominal obesity, hypertension and dyslipidaemia, which all contribute to the development of cardiometabolic diseases such as type 2 diabetes mellitus and cardiovascular diseases [1]. Over the past two decades, the global prevalence of people with the metabolic syndrome has considerably increased and has caused an alarming trend of increase in cardiometabolic diseases. Here, physical inactivity is one of the major contributing factors that highly correlates with mortality and hospitalization [2, 3].

Engaging in regular moderate-to-vigorous physical activity (MVPA) significantly improves cardiometabolic health and plays an important role in the prevention and management of cardiometabolic diseases. In this respect, the World Health Organization recommends practicing a weekly volume of 150–300 min at moderate intensity, 75–150 min at vigorous intensity or an equivalent combination of MVPA [4]. However, 28% of the adult and 80% of the adolescent population remains physically inactive [5, 6]. In addition, although research has mainly focussed on physical activity so far, it has become evident that sedentary behaviour, which is interdependent with time spent in MVPA, is also an important contributor to cardiometabolic disease development. Sedentary behaviour is defined as’any waking behaviour characterized by a low energy expenditure (≤ 1.5 metabolic equivalents), while being in a sitting or reclining posture’ [7]. Epidemiological and meta-analytic evidence has indicated that low levels of MVPA in combination with large volumes of sedentary behaviour are jointly associated with increased cardiometabolic morbidities and mortality in a dose-dependent manner [8–10]. Despite the detrimental health effects of prolonged sitting, adults still accumulate between 7.5 and 10 h of their day in sedentary pursuits during work, transportation and leisure time activities [10]. Although the 2020 guidelines on physical activity and sedentary behaviour encourage reducing periods of prolonged sitting, no specific strategy has been proposed to combat the negative effects of sedentary lifestyles [4]. Therefore, there is need for behaviour change strategies to reduce sedentary behaviour and to increase physical activity levels.

A variety of effective (multicomponent) behaviour change strategies, including environmental modifications, education, motivational counselling and technologies such as consumer wearable devices and smartphone applications have been applied to reduce sedentary behaviour in adults [11–15]. However, the majority of the current studies has focused on workplace interventions [11, 14] or assessed intervention effects using self-report [12]. In addition, most studies were insufficiently powered to detect significant improvements in cardiometabolic health [15]. Furthermore, systematic reviews mainly included multicomponent interventions, where the multicomponent character of these interventions limits separation of the effects of the individual components [11–13]. Interestingly, Compernolle et al. showed that self-monitoring-based behaviour change interventions are promising to reduce sedentary behaviours [16]. However, all included interventions consisted of multiple behaviour change techniques, making it impossible to determine whether the beneficial effects on sedentary behaviour were attributable to self-monitoring on itself, or to (a combination with) other behaviour change techniques. In this respect, self-monitoring with the aid of consumer wearable activity trackers (CWATs) could be a promising way to reduce sedentary behaviours. Here, CWATs are electronic wearable devices used for monitoring physical or health related metrics as physical activity, sedentary behaviour, heart rate (variability), and most of them are able to provide feedback on these parameters. These CWATs are consisted of pedometers (e.g. Omron and Yamax) and more sophisticated activity trackers such as Polar, Fitbit, Garmin and Apple Watch. It has already been shown that CWATs can effectively improve physical activity volumes [17–19]. However, interventions solely focusing on physical activity do not generally result in clinically meaningful reductions in sedentary time [13]. Nowadays, more sophisticated CWATs also implement information with regard to sedentary behaviour with the aid of providing alerts after prolonged sitting. In this respect, it may be possible that these CWATs are able to effectively reduce sedentary behaviour. In addition, since it is acknowledged that behaviour change benefits most from personalised coaching and from stimulating autonomy we expect that sedentary behaviour can be further reduced by adding motivational interviewing to CWAT-use. It has already been shown in systematic reviews [20, 21] and large randomised controlled trials [22, 23] that motivational interviewing is an effective way of building motivation and even recommended by the American Heart Association as an effective approach to promote physical activity and dietary changes [24]. However, limited evidence is available with regard to the effectiveness of motivational interviewing to reduce sedentary behaviour [25].

Therefore, this study aims to investigate the efficacy of a single component CWAT-only intervention and the added value of a multicomponent (CWATs + motivational interviewing) behaviour change intervention to reduce sedentary behaviour and increase physical activity within sedentary adults. The second aim is to investigate whether a reduction in sedentary behaviour also lead to improvements in cardiometabolic health.

## Research design and methods

### Subjects

Sedentary (sitting time of ≥ 9 h/day) healthy adults, or sedentary adults at risk to develop chronic diseases, aged between 40 and 75 years were recruited via online and paper advertisements. At risk participants were reported as having at least one of the following cardiometabolic risk factors: prehypertension (systolic blood pressure: 120–140 mmHg; diastolic blood pressure: 80–89 mmHg), overweight/obese (BMI: 25–35 kg/m^2^), hyperlipidaemia and/or prediabetes (HbA1c < 6.5%). Exclusion criteria were pregnancy, regularly (> 150 min per week during the last four months) being engaged in structured moderate-to-vigorous intensity physical exercise, any known contra-indication for physical activity, more than 14 alcohol consumptions per week, plans to follow a weight reduction programme with the aid of an energy restriction diet or a physical intervention programme during the study period, or participants diagnosed with any known metabolic disease. These criteria were assessed during a screening visit involving a medical examination, including their medical history, general health and medication use by means of a general health questionnaire. In addition, HbA1c was measured and the cardiovascular status was screened using a resting 12-lead electrocardiogram (Mortara ELI150c, Welch Allyn, Chicago, IL, USA) and resting blood pressure measurement (Omron M2, Omron Healthcare, Lake Forest, IL, USA). Throughout the study trial, participants were instructed to consume and maintain their habitual diet. All participants were informed in detail and were asked to provide written informed consent. The study was approved by the medical ethical committee of Hasselt University and was performed at Hasselt University (Diepenbeek, Belgium) between September 2018 and February 2021 in accordance with the principles of the Declaration of Helsinki. The recruitment period was between September 2018 and June 2020. The present study was registered at clinicaltrials.gov as NCT03853018.

### Study design

**Fig. 1:**
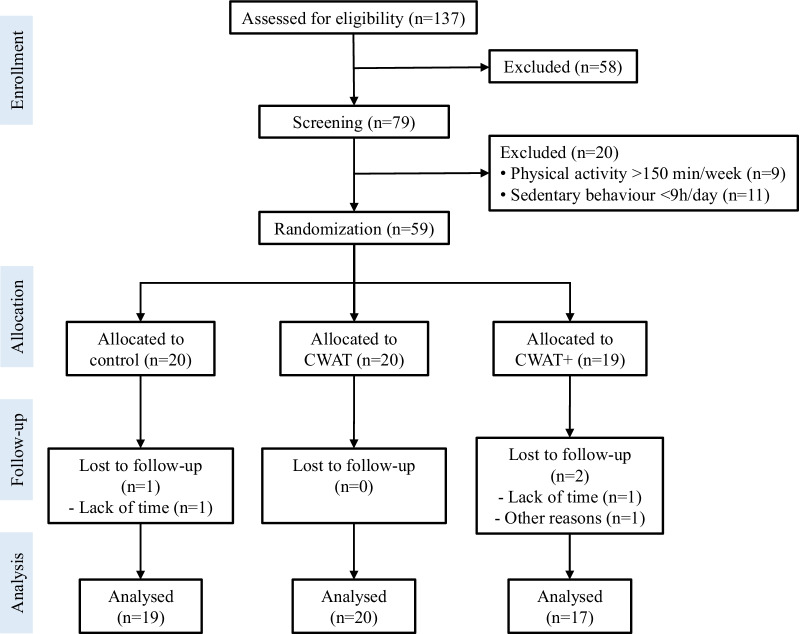
Study flow chart of the eligible and ultimately included participants

The study was carried out according to a three-armed, randomised controlled design. Prior to randomisation and baseline measurements, physical activity and sedentary behaviour was assessed with the aid of an accelerometer (activPAL3™, PAL Technologies Ltd, Glasgow, Scotland) for seven consecutive days to ensure that only sedentary participants were included. One week after the screening visit, eligible participants were included for baseline measurements. Participants were instructed to refrain from strenuous physical exercise three days before each test day and one day prior to each test day participants were requested not to consume alcohol. After a 12-h overnight fasting period prior to examination, all participants were refrained from consuming food, except for water ad libitum to prevent changes on biochemical analysis. First, anthropometry, body composition using dual energy X‐ray absorptiometry and blood pressure were assessed and venous blood samples were collected. Subsequently, an oral glucose tolerance test (OGTT) was performed to assess insulin sensitivity and beta cell function. Furthermore, dietary habits were assessed for seven consecutive days by means of a diary. Following baseline measurements, eligible participants were randomly assigned (Fig. 1) using an established allocation ratio of 1:1:1 to either 1: a group without any intervention (control group, CON), 2: a group receiving only an consumer wearable activity tracker (CWAT) or 3: a CWAT and additional motivational messages via the ELCIES (ELCIES, Gent, Belgium) lifestyle data platform (CWAT +). The control group was instructed to continue their habitual daily physical activity patterns and sedentary behaviours. The CWAT group received the Polar M200 activity tracker (Polar Electro, Kempele, Finland) and were assisted in creating a Polar flow account, downloading the application, synchronizing the Polar M200 with their individual Polar flow account and how to use the device. Participants received real-time feedback from the Polar device in terms of step count and inactivity alerts after 1 h of inactivity to break up sitting time to avoid prolonged sedentary behaviours. During the interruptions they were asked to walk or stand for several minutes. Participants were instructed to increase their step count to at least 10,000 steps per day, spread throughout the day during the interruptions of sedentary behaviour. Participants allocated to the CWAT + intervention also received the Polar M200. In addition, an initial personalized physical activity prescription was given, in accordance with the participants, based on baseline sedentary time, average number of steps per day and their occupational and daily physical activity levels. Here, the aim was to address sedentary behaviour via two target behaviours: replacing prolonged sitting with stepping of any intensity, or standing, and break up prolonged bouts of sedentary behaviour. Participants were supported by means of motivational interviewing under guidance of a clinical psychologist, which has been found to be an effective, relatively low intensity intervention whereby the interaction was mainly based on collaboration and autonomy [26]. This technique could be defined as a person-centred directive counselling style used to address individual ambivalence about behaviour change through placing the emphasis on individuals producing their own argument for change. Typical strategies to build motivation were goal-setting, a focus on self-efficacy, increasing beliefs about the positive health consequences of taking action (outcome expectancies), providing tips and tricks to decrease sedentary behaviour, motivate people to achieve their goal targets, probing for a rationale when participants did not reach their targets, and self-monitoring. The counselling sessions took place via chat conversations. The researchers contacted the participants from the CWAT + group on a weekly basis and individualized messages were framed positively and highlighted short-term outcomes specifically relating to social and mental health. In addition, the message content was tailored to the recipients. The frequency and content of chat conversations were tailored to the recipients, based on the needs and requests of the participant. For example, identification of personal reasons for reducing sedentary time, enforcing and introducing new identified benefits, identification of personal concerns for reducing sedentary time and discuss solutions, setting new goals and discussing possibilities to reduce sedentary time during occupational time or daily life. All conversations and visualization of physical activity information was supported via the messenger system of the ELCIES data platform (www.elcies.com). This secured platform promotes and supports a healthy lifestyle including to address prolonged sitting and physical inactivity. Once the ultimate goal of 10,000 steps per day was reached participants were encouraged to maintain this level of physical activity. Furthermore, in addition to motivational interviewing, at the start of the intervention period participants of the CWAT + group got an information session to increase participants’ awareness of the negative independent impact of sedentary behaviour on the risk of chronic disease development. Here, it was important that participants became aware that interrupting sedentary time was important during the day, next to performing physical activity. We considered intervention adherence as successful when any increase in total step count and/or postural transitions above each participant’s own average baseline levels.

Blocked randomisation was performed by an independent researcher using a random block size of two, three or four with the aid of sealed envelopes. Hereafter, participants were enrolled into a 12-week intervention period. Following 12 weeks of either CON, CWAT or CWAT + intervention baseline measurements were repeated.

### Physical activity and sedentary behaviour assessment

Physical activity and body postures were quantified using the activPAL3™ activity monitor (PAL Technologies Ltd, Glasgow, Scotland), a triaxial accelerometer and inclinometer, as previously described [27]. The device was enclosed with a nitrile sleeve and attached to the anterior mid-thigh of the participants right leg using an adhesive dressing (Tegaderm, 3 M, Minnesota, USA). Participants wore the device for a period of 7 consecutive days and 24 h hours per day, without removing it at any time. The activPAL3™ could accurately measure posture allocation and free-living physical activity. Raw data were processed and analysed using the PALanalysis software (version 8, PAL Technologies Ltd., Glasgow, Scotland). Sleeping and waking hours on each wear day were identified using the built-in algorithm that automatically detected sleeping time [28]. Data from the ActivPAL software were also processed using customised software written in MATLAB R2013b (MathWorks, Natick, MA, USA) to adjust for incorrect sleeping times [29]. Output variables from the ActivPAL software included sleeping time and waking time which was consisted of sedentary time (sitting or lying) as primary outcome, standing time and physical activity including step count and step cadence (low intensity physical activity [< 100 steps/min] and MVPA [> 100 steps/min]) [28, 30]. Furthermore, short (< 30 min) and prolonged (> 60 min) sedentary bouts were measured.

### Anthropometry and body composition

Body height was measured to the nearest 0.1 cm using a wall-mounted Harpenden stadiometer, with participants barefoot. Body weight (in underwear) was determined using a digital-balanced weighting scale to the nearest 0.1 kg. BMI was calculated from weight and height measurements (weight/height^2^). Waist and hip circumferences was measured to the nearest 0.1 cm using a flexible metric measuring tape with participants barefoot (in underwear) in standing position. Waist circumference was measured at the midpoint between the lower rib margin and the top of the iliac crest. Hip circumference was measured at the widest circumference of the hip at the level of the greater trochanter. Both measures were assessed in triplicate and the mean value of the triplicate measurements was used in the analysis. Waist-to-hip ratio was calculated by dividing waist circumference (cm) by hip circumference (cm). Whole body fat, lean tissue mass and bone mineral density were evaluated using Dual Energy X-ray Absorptiometry (Hologic Series Delphi-A Fan Beam X-ray Bone Densitometer, Vilvoorde, Belgium).

### Blood pressure

After an initial resting period of 20 min with participants in a supine position in a quiet room with constant temperature (21 °C), blood pressure (BP) was measured at least 3 times at 2-min intervals until blood pressure was stabilized using an electronic sphygmomanometer (Omron^®^, Omron Healthcare, IL, USA) from the left arm and documented as the mean value of the 3 final measurements. Mean arterial pressure (MAP) was calculated as MAP = systolic BP + (2 × diastolic BP)/3.

### Blood glucose, insulin and serum lipids

After antecubital catheter placement, fasting blood samples were obtained for the measurement of cardiometabolic risk factors. Serum separation and sodium fluoride (NaF) containing BD vacutainer™ tubes (Becton, Dickinson and Company, Franklin lakes, NY, USA) were collected. To obtain plasma, NaF tubes were immediately centrifuged at 1300×*g* for 15 min. Serum tubes coagulated for at least 30 min prior to centrifuging at 1300×*g* for 15 min. All centrifugation steps were performed at room temperature (21 °C). Supernatants were immediately portioned into aliquots and frozen at − 20 °C and subsequently moved to a − 80 °C freezer until analysis at the end of the trial. Glucose, insulin, total cholesterol, high-density lipoprotein cholesterol, low-density lipoprotein cholesterol and triglycerides concentrations were automatically assessed on the Roche Cobas 8000 (Roche Diagnostics International Ltd, Rotkreuz, Switzerland). Sodium heparinized 18 µl capillary tubes (Marienfeld GmbH, Lauda-Königshofen, Germany) were used to collect capillary blood from the middle finger. Blood glycated haemoglobin A1c (HbA1c) concentration was assessed using ion exchange chromatography (Menarini HA-8180 HbA1c auto-analyser, Menarini Diagnostics, Diegem, Belgium).

### Insulin sensitivity and beta cell function

A standard 5-point oral glucose tolerance test (OGTT) was performed for assessment of whole body/tissue specific insulin sensitivity and beta cell function. Participants ingested a solution (250 ml) containing 75 g dextrose, and venous blood samples were obtained at t = 0, 30, 60, 90 and 120 min for assessment of venous glucose and insulin concentration. From glucose and insulin concentrations, homeostatic model assessment for insulin resistance was calculated by: fasting glucose (mmol/l) × fasting insulin (µU/ml)/22.5 [31]. In addition, whole-body insulin sensitivity was estimated using the Matsuda index and calculated as: 10,000/√[fasting glucose (mg/dl) × fasting insulin (µU/ml)) × (mean glucose during OGTT (mg/dl) × mean insulin during OGTT (µU/ml)] [32]. The quantitative insulin sensitivity check index was calculated as: 1/log(fasting insulin (µU/ml) + log(fasting glucose (mg/dl) [31]. Beta cell function was estimated by calculation of the insulinogenic index (IGI) by: ratio of increment of insulin (µU/ml) and glucose (mg/dl) in the first 30 min of OGTT [31]. The total area under the curve (tAUC) for glucose and insulin for the 2 h period is calculated using the trapezoidal rule [33]. Tissue specific insulin resistance was calculate using the hepatic insulin resistance index and the muscle insulin resistance index. The hepatic insulin resistance index was calculated as the product of the tAUCs for glucose and insulin during the first 30 min of the OGTT (glucose0–30[tAUC in mg/dl h] × insulin 0–30[tAUC in µU/ml h]) and the muscle insulin resistance index was calculated as the rate of decay of glucose concentration during the OGTT divided by the mean insulin concentration during the OGTT in mg/dl/min/µU/ml)*.* The rate of decay was calculated as the slope of the least square fit to the decline in glucose concentration from peak to nadir, as described by Vogelzangs et al. [34].

### Energy and nutrient intake assessment

Habitual dietary intake was assessed using a self-administered food diary at the start and after the 12-week intervention period. Participants recorded all food and beverages consumed over seven consecutive days and from this the total caloric intake and macronutrient content was calculated.

### Statistical analysis

Statistical analyses were performed by IBM SPSS® version 27.0 (IBM SPSS Statistics for Windows, Chicago, IL, USA). Data were expressed as mean ± SD, unless otherwise indicated.

Shapiro–Wilk test was used to test normality of the data (p < 0.05). Natural log transformation was performed if the outcome was not normally distributed. Data were analysed using an intention-to-treat approach. Differences in response between groups were analysed using general linear model analyses with the difference between baseline and 12-week intervention as dependent variable, group (control, CWAT and CWAT +) as fixed factor and baseline values of the outcome variables as covariates. Linear mixed models were used to assess whether there were differences in insulin and glucose concentration during OGTT. First, the difference between baseline and after the 12-week intervention period for each separate time point of the OGTT were calculated. Then, an interaction effect was evaluated, where group (control, CWAT and CWAT +) was a between-subjects factor, and time (five different time points of the OGTT) was a within-subjects factor. A pairwise analysis (Bonferroni post-hoc comparison test) was performed when the between-subjects factor was statistically significant. A p-value < 0.05 (2-tailed) was considered statistically significant. Associations between physical activity intensities and cardiometabolic health were performed using multivariate linear regression models. Metabolic variables that were significant different between groups, including body mass, waist circumference, fat mass, insulin concentration after 120 min of the OGTT, tAUC of insulin and triglyceride concentration, were included as dependent variables. Independent variables were change in standing time, LPA and MVPA over the 12-week intervention period. Model 1 was the unadjusted model, model 2 corrected for potential confounders sex, age, body height, smoking status, chronic disease, medication, food intake and baseline measurements of standing, LPA and MVPA and model 3 also corrected for all other variables (standing, LPA and/or MVPA).

The sample size calculation was performed using GPower v. 3.1 (Düsseldorf, Germany). Prince et al. showed in a systematic review and meta-analysis a significant reduction in sedentary behaviour (effect size d: 1.08) in adults [13]. Based on a statistical power > 0.8 and a two-sided alpha of 0.017 (0.05/3 groups) it was calculated that a sample size of 15 individuals per group had to be included in the present study. To prevent insufficient power to detect significant improvements in cardiometabolic health, a second sample size calculation was performed. Using the same values as stated above and an effect size of 0.85 (based on observed triglyceride concentrations from Duvivier et al*.* [35]), it was calculated that a sample size of 18 participants per group had to be included in the present study. Taking into account a drop-out rate of 10%, the number of participants to include in this study was at least 18 participants per group, resulting in a final sample size of 60 participants.

## Results

137 participants were initially assessed for study entry of which 79 individuals were effectively screened. From these 79 individuals, 20 participants were excluded due to a sedentary time < 9 h/day (n = 11) or spending more than 150 of structured physical activity (n = 9). These 59 individuals were randomly assigned to either the control (n = 20), CWAT (n = 20) or CWAT + (n = 19) group. Three participants from both the control (n = 1) and the CWAT + (n = 2) group were dropped out mainly due to lack of time (Fig. 1). The age range of the participants was between 41 and 71 years (53.3 ± 8.7 years) and the BMI ranged between 19.7 and 34.8 kg/m^2^ (26.0 ± 4.1 kg/m^2^). In addition, the total population consisted of 21 (36%) males and 38 (64%) females. In total 9 participants were included with chronic diseases, of which 3 with chronic respiratory diseases (con: n = 1, CWAT + : n = 2) and 6 with cardiovascular diseases (CWAT: n = 5, CWAT + : n = 1). At baseline, participants spent 34% (8.1 ± 0.6 h) of the day sleeping, 45% (10.8 ± 1.2 h) of their waking hours sedentary, 15% (3.6 ± 1.0 h) in standing activities and only 5% (1.1 ± 0.3 h) in LPA and 2% (0.4 ± 0.2 h) in MVPA. No significant differences for all variables were found between groups at baseline.

### Physical activity and sedentary behaviour

**Fig. 2:**
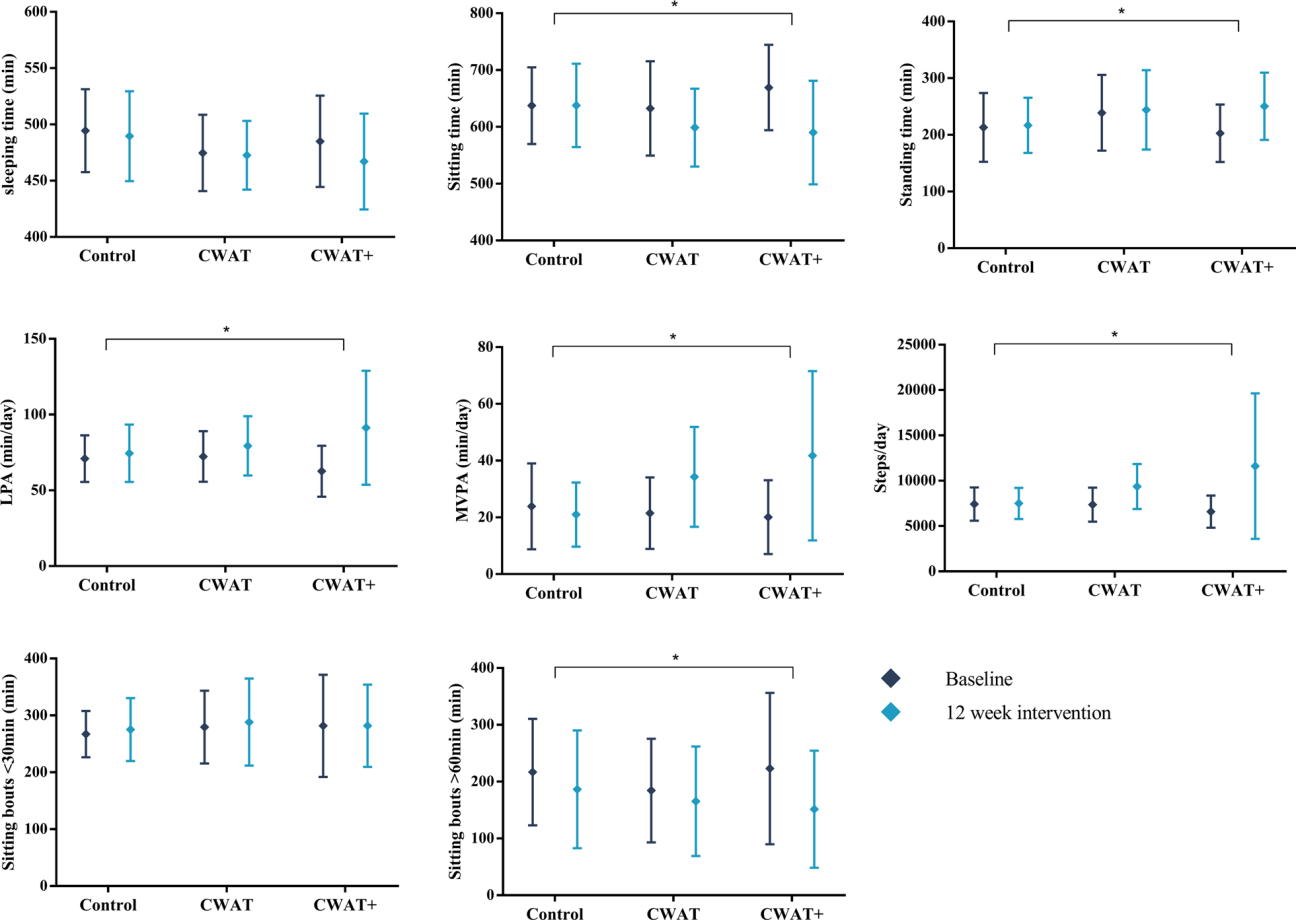
Average time spent in sleeping, different physical activity intensities and sedentary behaviours before and after a 12-week CWAT-based intervention period within the control, CWAT and CWAT + groups. Data are presented as means ± standard deviations. * *p* < 0.05 of mean difference (12 weeks—baseline) between groups. *LPA* light intensity physical activity, *MVPA* moderate-to-vigorous intensity physical activity, *CWAT* consumer wearable activity tracker

After the 12-week intervention period and compared with the control group, participants from the CWAT + group significantly increased their step count (+ 3301 ± 3558 vs. + 184 ± 2045 steps/day; *p* = 0.036), LPA (+ 32 ± 34 vs. + 3 ± 24 min/day; *p* = 0.040) and MVPA (+ 20 ± 28 vs. − 2 ± 13 min/day; *p* = 0.005) (Fig. 2). In addition, standing time was increased (+ 64 ± 57 vs. + 2 ± 52 min/day; *p* = 0.015), whereas the time spent in sedentary behaviours was significantly reduced (− 79 ± 89 vs. + 2 ± 61 min/day), mainly due to spending less time in bouts of more than 1 h (− 85 ± 68 vs. − 29 ± 97 min/day; *p* = 0.024) in CWAT + when compared to the control group. In contrast, no significant improvements with respect to sitting time and physical activity within the CWAT group were observed, compared to the control and CWAT + group.

### Anthropometrics and body composition

The 12-week intervention significantly decreased body weight (CWAT + : − 1.6 ± 3.1 kg, CWAT: + 0.3 ± 1.0 kg; *p* = 0.021, Con: + 0.6 ± 2.1 kg; *p* = 0.009) and body mass index (CWAT + : − 0.5 ± 1.0 kg/m^2^, CWAT: + 0.1 ± 0.3 kg/m^2^; *p* = 0.017, Con: + 0.2 ± 0.7 kg/m^2^; *p* = 0.011) within the CWAT + population, compared to the CWAT and control group (Table 1). Waist circumference was decreased in both the CWAT (− 1.1 ± 2.6 cm vs. + 1.7 ± 4.1 cm; *p* = 0.049) and CWAT + (− 2.3 ± 4.0 cm vs. + 1.7 ± 4.1 cm; *p* = 0.004) group compared to the controls, whereas only participants from the CWAT + group significantly reduced their hip circumference (CWAT + : − 2.0 ± 4.8 cm, CWAT: − 1.1 ± 3.7 cm; *p* = 0.952, Con: + 1.4 ± 3.3 cm; *p* = 0.014), body fat mass (CWAT + : − 1.3 ± 2.6 kg, CWAT: + 0.1 ± 1.0 kg; *p* = 0.095, Con: + 0.4 ± 1.6 kg; *p* = 0.021) and percentage body fat mass (CWAT + : − 1.4 ± 2.4%, CWAT: + 0.1 ± 1.3%; *p* = 0.124, Con: + 0.2 ± 1.2%; *p* = 0.026) compared to the control group.

**Table 1:** Subject characteristics before and after a 12-week CWAT-based intervention period within the control, CWAT and CWAT + groups

General features	Control		CWAT		CWAT +		Intervention effects		
	Baseline (n = 20)	12 weeks (n = 19)	Baseline (n = 20)	12 weeks (n = 20)	Baseline (n = 19)	12 weeks (n = 17)	CWAT vs control	CWAT + vs. control	CWAT + vs. CWAT
Age (years)	53.8 ± 8.5	54.2 ± 8.8	52.4 ± 8.7	52.8 ± 8.7	53.8 ± 9.2	54.7 ± 9.5			
Sex (m/f)	6/14	6/13	7/13	7/13	8/11	7/10			
Body weight (kg)	75.8 ± 11.5	77.2 ± 11.6	76.6 ± 14.6	76.9 ± 14.7	74.7 ± 12.7	72.6 ± 12.5	− 0.28 (− 2.01, 1.46)	− 2.19 (− 4.00, − 0.37)*	− 1.91 (− 3.70, − 0.12)*
Body height (cm)	169.7 ± 7.9	170.4 ± 7.3	171.1 ± 7.9	171.1 ± 8.0	171.3 ± 10.3	170.2 ± 10.2			
BMI (kg/m^2^)	26.3 ± 3.8	26.6 ± 4.1	26.2 ± 4.9	26.3 ± 4.9	25.4 ± 3.6	25.0 ± 3.6	− 0.06 (− 0.65, 0.52)	− 0.72 (− 1.33, − 0.14)*	− 0.66 (− 1.26, − 0.06)*
Waist circumference (cm)	88.5 ± 10.0	90.9 ± 10.7	89.2 ± 13.9	88.0 ± 14.3	87.2 ± 10.1	85.1 ± 9.2	− 2.87 (− 5.72, − 0.01)*	− 4.11 (− 7.09, − 1.13)*	− 1.24 (− 4.20, 1.70)
Hip circumference (cm)	98.2 ± 9.3	100.2 ± 10.4	99.7 ± 11.2	98.6 ± 11.4	97.4 ± 8.0	95.6 ± 7.2	− 2.46 (− 5.60, 0.67)	− 3.48 (− 6.75, − 0.21)*	− 1.02 (− 4.25, 2.22)
Waist-to-hip-ratio	0.90 ± 0.08	0.91 ± 0.08	0.89 ± 0.08	0.89 ± 0.10	0.90 ± 0.07	0.89 ± 0.07	-0.01 (− 0.04, 0.02)	− 0.01 (− 0.04, 0.02)	− 0.01 (− 0.04, 0.03)
Lean mass (kg)	46.5 ± 7.1	47.3 ± 6.5	48.3 ± 6.5	48.6 ± 8.6	47.9 ± 9.1	47.3 ± 9.2	0.21 (− 0.73, 1.11)	− 0.36 (− 1.35, 0.63)	− 0.57 (− 1.55, 0.41)
Fat mass (kg)	26.0 ± 9.4	26.5 ± 10.0	24.5 ± 9.9	24.6 ± 10.1	23.1 ± 6.2	21.9 ± 7.3	− 0.26 (− 1.70, 1.18)	− 1.67 (− 3.21, − 0.14)*	− 1.41 (− 3.21, − 0.14)
Fat mass (%)	34.0 ± 9.6	33.9 ± 9.9	31.9 ± 9.5	31.8 ± 9.4	31.5 ± 6.6	30.4 ± 8.0	− 0.3 (− 1.6, 1.1)	− 1.5 (− 2.9, − 0.1)*	− 1.3 (− 2.6, 0.2)
Energy intake (kcal)	1503 ± 255	1600 ± 450	1726 ± 348	1793 ± 264	1635 ± 351	1595 ± 630	− 87 (− 330, 505)	− 120 (− 557, 317)	− 33 (− 437, 371
Fat (g)	53 ± 16	56 ± 23	64 ± 19	65 ± 11	58 ± 14	60 ± 26	− 6 (− 23, 19)	− 4 (− 27, 19)	2 (− 19, 23)
Protein (g)	71 ± 15	76 ± 20	68 ± 18	74 ± 22	73 ± 18	68 ± 24	4 (− 22, 31)	− 10 (− 37, 17)	− 14 (− 40, 11)
Carbohydrate (g)	170 ± 30	179 ± 42	196 ± 42	208 ± 40	189 ± 44	180 ± 75	− 8 (− 49, 34)	− 10 (− 54, 33)	− 3 (− 43, 38)
HbA1c (%)	5.3 ± 0.3		5.4 ± 0.3		5.3 ± 0.4				
Smoking status (n)									
Never	0		0		1				
Former	5		7		3				
Never	15		13		15				
Chronic disease (n)									
Respiratory	1		0		2				
Cardiovascular	0		5		1				
Medication (n)									
Beta blocker	0		3		2				
Angiotensin II-antagonist	0		2		1				
Bronchodilator	1		0		1				

Data are expressed as mean ± SD

*BMI* body mass index

*p < 0.05. The intervention effects are mean changes (95% CI) obtained from general linear model analyses with baseline value as covariate

### Lipid profile and insulin sensitivity

**Fig. 3:**
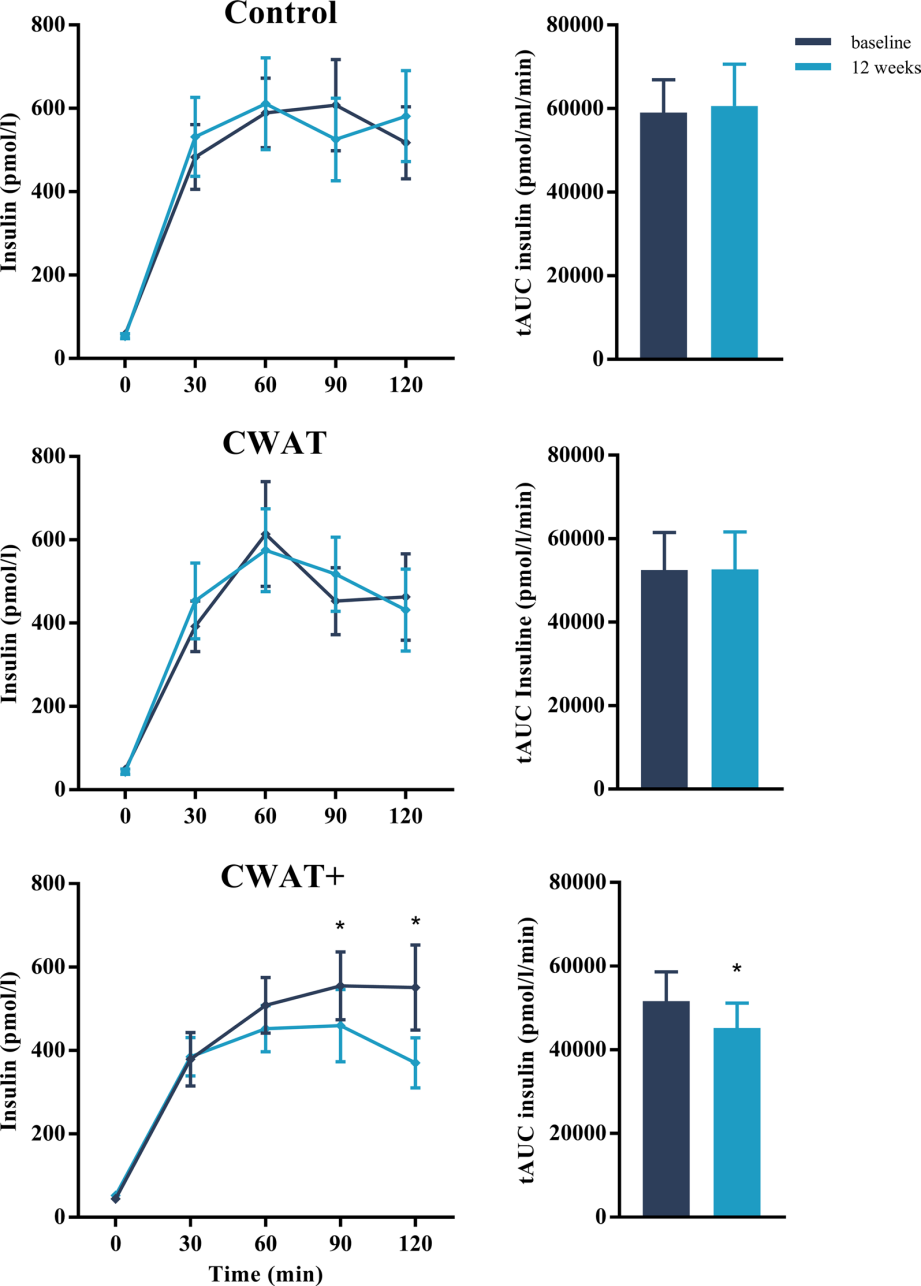
Insulin concentrations during a 2-h oral glucose tolerance test (left hand panel) and the average area under the curve (right hand panel) for each individual group (control, CWAT and CWAT +) at baseline and after a 12-week intervention period. Data are presented as means ± standard errors of the mean. *p < 0.05 of mean difference (12 weeks—baseline) between groups. *tAUC* total area under the curve, *CWAT *consumer wearable activity tracker

With respect to the lipid profile only triglyceride concentrations were significantly decreased in the CWAT + group (− 0.31 ± 0.43 mmol/l; *p* = 0.043) compared to the control group (− 0.05 ± 0.33 mmol/l) after a 12-week intervention period, while no differences were found between the CWAT group and the control group (Table 2). In addition, the insulin concentration (CWAT + : − 145 ± 212 pmol/l, Con: 30 ± 301 pmol/l; *p* = 0.021) after 2 h of the OGTT and the quantitative insulin sensitivity check index (CWAT + : − 0.02 ± 0.02, Con: 0.01 ± 0.02; *p* = 0.010) significantly improved in the CWAT + group, compared to the control group. A significant between-subject difference (*p* = 0.039) of the tAUC of insulin concentration was found within the CWAT + group (baseline: 51,613 ± 29,688 mmol/l min vs. 12 weeks: 45,233 ± 23,636 mmol/l min) compared to the control (baseline: 59,012 ± 34,473 mmol/l min vs. 12 weeks: 60,633 ± 39,986 mmol/l min, Fig. 3) and CWAT groups (baseline: 52,514 ± 38,236 mmol/l min vs. 12 weeks: 52,649 ± 36,907 mmol/l min). In addition, linear mixed models revealed a significant between-subject (*p* = 0.029), within subject (*p* = 0.037) and interaction effect (*p* = 0.045) of insulin concentrations during the OGTT. Post-hoc comparison test showed significant between group differences at 90 (con: − 9 ± 185 pmol/l; *p* = 0.063, CWAT: 53 ± 205 pmol/l; *p* = 0.017, CWAT + : − 78 ± 173 pmol/l) and 120 min (con: 30 ± 301 pmol/l; *p* = 0.039, CWAT: 27 ± 217 pmol/l; *p* = 0.043, CWAT + :− 151 ± 218 pmol/l) of the OGTT.

**Table 2:** Cardiometabolic risk factors and parameters before and after a 12-week CWAT-based intervention period within the control, CWAT and CWAT + groups

	Control		CWAT		CWAT +		Treatment effects		
	Baseline (n = 20)	12 weeks (n = 19)	Baseline (n = 20)	12 weeks (n = 20)	Baseline (n = 19)	12 weeks (n = 17)	CWAT vs. control	CWAT + vs. control	CWAT + vs. CWAT
Cardiovascular health									
Systolic BP (mm Hg)	120 ± 13	117 ± 12	123 ± 12	120 ± 12	124 ± 12	125 ± 15	1 (− 6, 8)	4 (− 3, 12)	3 (− 4, 10)
Diastolic BP (mm Hg)	79 ± 8	77 ± 7	83 ± 9	80 ± 8	81 ± 8	81 ± 9	0 (− 4, 4)	3 (− 1, 6)	3 (− 1, 6)
Mean arterial pressure (mm Hg)	93 ± 10	90 ± 8	96 ± 9	93 ± 9	95 ± 9	96 ± 10	0 (− 4, 5)	3 (− 1, 8)	3 (− 2, 7)
Resting heart rate (bpm)	62 ± 6	61 ± 7	59 ± 9	60 ± 8	57 ± 7	60 ± 8	1 (− 4, 6)	3 (− 2, 8)	2 (− 3, 6)
Total cholesterol (mmol/l)	5.00 ± 0.93	4.80 ± 0.91	5.40 ± 1.12	4.78 ± 0.88	5.67 ± 1.27	4.64 ± 0.98	− 0.17 (− 0.79, 0.45)	− 0.41 (− 1.06, 0.24)	− 0.24 (− 0.87, 0.39)
HDL cholesterol (mmol/l)	1.39 ± 0.24	1.26 ± 0.33	1.62 ± 0.49	1.36 ± 0.23	1.61 ± 0.51	1.26 ± 0.29	0.07 (− 0.14, 0.28)	− 0.05 (− 0.25, 0.18)	− 0.11 (− 0.31, 0.10)
LDL-cholesterol (mmol/l)	3.61 ± 0.82	3.54 ± 0.81	3.78 ± 1.10	3.42 ± 0.90	4.06 ± 0.98	3.38 ± 0.82	− 0.20 (− 0.67, 0.27)	− 0.38 (− 0.87,0.11)	− 0.18 (− 0.67, 0.31)
Triglycerides (mmol/l)	0.98 ± 0.41	1.05 ± 0.55	1.25 ± 0.71	1.02 ± 0.57	1.22 ± 0.38	0.94 ± 0.37	− 0.25 (− 0.55, 0.05)	− 0.31 (− 0.62, − 0.01)*	− 0.07 (− 0.63, 0.23)
Glucose tolerance									
Fasting glucose (mmol/l)	5.3 ± 0.5	5.2 ± 0.5	5.1 ± 0.7	5.2 ± 0.5	5.3 ± 0.4	5.5 ± 1.0	0.3 (− 0.5, 0.5)	0.2 (− 0.3, 0.7)	0.2 (− 0.3, 0.7)
Fasting insulin (pmol/l)	60 ± 31	55 ± 24	54 ± 35	46 ± 29	44 ± 28	52 ± 17	− 5 (− 20, 10)	5 (− 11, 21)	10 (− 5, 25)
Glucose 120 min (mmol/l)	6.5 ± 1.8	7.3 ± 2.3	6.2 ± 1.7	6.4 ± 1.9	6.9 ± 1.7	6.6 ± 1.5	− 0.3 (− 1.6, 0.9)	− 0.6 (− 1.9, 0.7)	− 0.3 (− 1.6, 1.0)
Insulin 120 min (pmol/l)	547 ± 389	571 ± 425	490 ± 429	421 ± 396	551 ± 433	371 ± 241	− 68 (− 266, 129)	− 173 (− 375, − 29)*	− 104 (− 304, 95)
Glucose tAUC (mmol/l min)	862 ± 159	903 ± 209	832 ± 155	845 ± 196	890 ± 139	862 ± 180	− 2 (− 132, 127)	− 60 (− 192, 72)	− 57 (− 194, 79)
Matsuda index	5.04 ± 3.42	5.45 ± 3.51	6.07 ± 3.71	7.13 ± 4.59	5.75 ± 2.37	5.18 ± 1.72	0.27 (− 1.70, 2.24)	− 0.65 (− 2.70, 1.40)	− 0.91 (− 2.94, 1.11)
IGI	209 ± 151	178 ± 109	173 ± 155	183 ± 165	113 ± 201	156 ± 81	− 9 (− 53, 71)	6 (− 59, 72)	− 3 (− 67, 62)
QUICKI	0.35 ± 0.03	0.36 ± 0.04	0.36 ± 0.04	0.37 ± 0.04	0.37 ± 0.03	0.35 ± 0.02	0.01 (− 0.01, 0.03)	− 0.02 (− 0.04, 0.00)*	− 0.03 (− 0.05, 0.01)*
HOMA-IR	2.07 ± 1.16	1.88 ± 0.93	1.86 ± 1.40	1.55 ± 1.07	1.54 ± 1.11	1.90 ± 0.91	− 0.19 (− 0.79, 0.42)	0.30 (− 0.34, 0.93)	0.49 (− 0.14, 1.11)
mISI	0.11 ± 0.08	0.13 ± 0.1	0.14 ± 0.10	0.18 ± 0.19	0.14 ± 0.08	0.16 ± 0.08	0.03 (− 0.09, 0.14)	0.03 (− 0.09, 0.15)	0.00 (− 0.12, 0.12)
HIRI	32.8 ± 11.0	34.0 ± 13.0	32.2 ± 13.0	31.7 ± 13.5	27.7 ± 10.7	30.1 ± 6.7	− 1.0 (− 7.2, 5.2)	− 0.8 (− 7.3, 5.7)	0.2 (− 6.2, 6.6)

Data are expressed as mean ± SD

*BP* blood pressure, *bpm* beats per minute, *HDL* high-density lipoprotein, *LDL* low-density lipoprotein, *AUC* area under curve, *IGI* insulinogenic index, *QUICKI* quantitative insulin sensitivity check index, *HOMA-IR* homeostatic model assessment of insulin resistance, *mISI* muscle insulin sensitivity index, *HIRI* hepatic insulin resistance index

*p < 0.05. The intervention effects are mean changes (95% CI) obtained from general linear model analyses with baseline value as covariate

### Associations between cardiometabolic risk, sedentary behaviour and physical activity

**Fig. 4:**
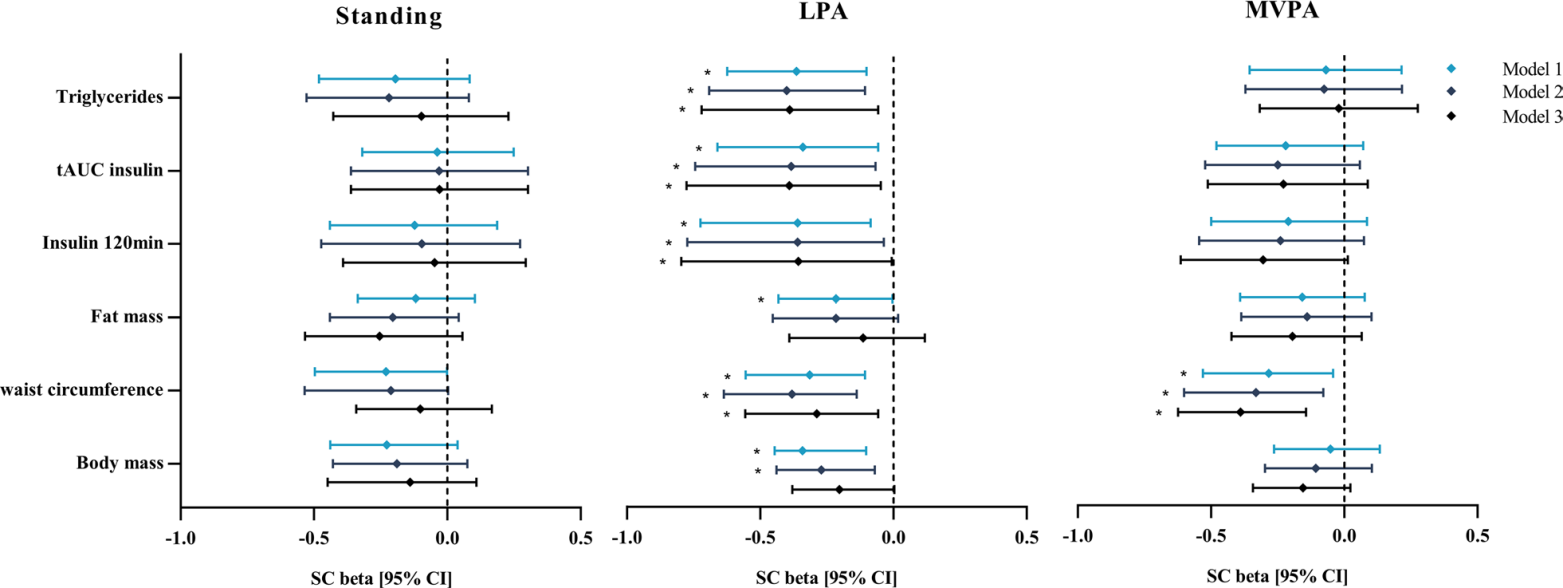
Multivariate regression analyses for the associations between standing, LPA, MVPA and different cardiometabolic health outcomes. Model 1: unadjusted; model 2: adjusted for covariates sex, body height, smoking status, chronic disease, medication, food intake and baseline measurements of standing, LPA and MVPA; model 3: adjusted for all covariates from model 2 and standing, LPA and/or MVPA. Data are presented as standardized coefficient of beta [95% confidence interval]. *p < 0.05. *LPA* light intensity physical activity, *MVPA* moderate-to-vigorous intensity physical activity, *HDL* high-density lipoprotein, *tAUC* total area under the curve

An increase in LPA was associated with reduced body weight (SC β = − 0.341 [− 0.445 to − 0.103]; r^2^ = 0.169; *p* = 0.002), waist circumference (SC β = − 0.314 [− 0.554 to − 0.107]; r^2^ = 0.147; *p* = 0.005), fat mass (SC β = − 0.216[− 0.432 to − 0.003]; r^2^ = 0.076; *p* = 0.002), insulin concentration after 2 h of OGTT (SC β = − 0.360 [− 0.724 to − 0.086]; r^2^ = 0.104; *p* = 0.014), tAUC insulin (SC β = − 0.340 [− 0.660 to − 0.057]; r^2^ = 0.115; *p* = 0.021) and triglyceride concentrations (SC β = − 0.363 [− 0.623 to − 0.101]; r^2^ = 0.132; *p* = 0.007). However, after adjusting for all covariates only waist circumference (SC β = − 0.287 [− 0.556 to − 0.057]; r^2^ = 0.391; *p* = 0.001), insulin concentration after 2 h of OGTT (SC β = -0.369 [− 0.821 to − 0.010]; r^2^ = 0.199; *p* = 0.045), tAUC insulin (SC β = − 0.395 [− 0.774 to − 0.059]; r^2^ = 0.230; *p* = 0.024) and triglyceride concentration (SC β = -0.367 [− 0.688 to − 0.043]; r^2^ = 0.230; *p* = 0.027) remain statistically associated with increased LPA levels (Fig. 4). In addition, an increased MVPA was only associated with a lower waist circumference (SC β = − 0.370 [− 0.574 to − 0.103]; r^2^ = 0.342; *p* = 0.006) when adjusted for all covariates.

## Discussion and conclusions

The aim of the current study was to investigate the efficacy of a single component CWAT-only intervention and the added value of a multicomponent (CWATs + motivational interviewing) behaviour change intervention to reduce sedentary behaviour and increase physical activity within sedentary adults. Here, we found that the multicomponent intervention (CWAT + : CWAT-use + motivational interviewing) significantly reduced sedentary behaviour and increased physical activity, whereas the single component (CWAT-only) intervention did not. Moreover, the reduction of sedentary behaviour within the CWAT + group was accompanied by an improvement in cardiometabolic health variables such as reduced body weight, waist circumference, fat mass, triglyceride concentration and enhanced insulin sensitivity. In addition, most favourable effects were found when LPA was increased instead of standing or MVPA.

Participants from the CWAT-group were not provided with behavioural change strategies, tips and tricks to decrease sedentary behaviour and information regarding the health consequences of prolonged sedentary behaviour. Therefore, these additional motivational techniques seem to be essential for improving both sedentary time and physical activity. This was also confirmed by Gardner et al*.* who showed that self-monitoring, goal setting, information and education on health consequences and motivational counselling were important components to reduce sedentary behaviours [36]. Important to mention is that we could not explore what component was most beneficial for reducing sedentary behaviour. Therefore, the significant differences could be due to the motivational counselling, the information session or both, as it has been shown by Gardner et al. that both education and behaviour change techniques are important to beneficially affect behaviours [36]. These techniques could be a reason why participants from the CWAT-group were not able to reduce their sedentary behaviours. Although it is known that CWATs could be effective tools to improve physical activity levels [18], Martin et al*.* showed that beneficial effects were observed for interventions specifically targeting the reduction in sedentary time instead of interventions in which combined approaches (reducing sedentary behaviour and increasing physical activity levels) were used [12]. Indeed, in the current study, participants from the CWAT-only group only focused on their step count (physical activity intervention). The CWATs used in this study provided information on step count and gave cues for inactivity when participants sat for more than one hour. Although the average step count from the CWAT group was increased (although not significant), a lot of inactivity stamps were observed within this group. Therefore, we could assume that they focused more on physical activity (step count) rather than sedentary behaviour. In contrast, CWAT + participants were mainly focused on reducing their sedentary time due to behavioural changes. Participants from the CWAT + group significantly reduced their daily sitting time by almost 80 min, which are in line with a systematic review from Prince et al*.* [13]*.* They focused on both sedentary behaviour and physical activity interventions and found that interventions with a focus on physical activity produced less consistent findings and generally resulted in modest reductions in sedentary time compared to interventions solely targeting sedentary behaviour. Therefore, although CWATs contain behaviour change strategies, including social support and providing feedback to increase physical activity, more attention needs to be paid on reducing sedentary behaviour to bring this into conscious awareness.

Furthermore, the specific goal setting approach also affects the decrease of sedentary behaviour. Here, participants from the CWAT-group were instructed to reach a daily step count of 10,000 steps per day, which probably led to a lower self-efficacy to achieve this goal. In contrast, CWAT + participants made their own goals to reduce sedentary behaviour and increase physical activity levels, possibly leading to a higher self-efficacy and motivation. Due to these self defined goals, the step count varied substantially from each other and may explain the high dispersion of the physical activity variables such as MVPA and LPA within the CWAT + group.

These results are confirmed by Qiu et al*.* who showed that setting an alternative personalized step goal (used in the CWAT + group) yields significantly reduced sedentary behaviours among participants with a CWAT-device instead of a goal of 10,000 steps/day, which were not able to significantly reduce sedentary time [37]. This means that setting small specific own goals are an important part of motivating individuals to reduce their sedentary behaviour.

The reduction in sedentary time and increment in standing and physical activity (LPA and MVPA) resulted in significant improvements in cardiometabolic health outcomes. These findings were consistent with a recent meta-analysis of Hadgraft et al*.* who showed beneficial effects on body weight, percentage body fat, waist circumference, insulin sensitivity and lipid metabolism after sedentary behaviour interventions in free-living conditions [11]. In addition, isotemporal substitution analyses indicated beneficial cardiometabolic health effects with the reallocation of 30 min per day of sedentary time with equal time of either LPA or MVPA, suggesting clinically meaningful [38]. These improvements were consistent with our results, especially in terms of triglycerides and insulin sensitivity. We found a significant decrease in anthropometrics, of which waist circumference (CWAT + vs. control: − 2 cm; − 2.4%) is most important in clinical practice, and triglyceride concentration (CWAT + vs. control: − 0.31 mmol/l). It has been shown that these reductions have clinical significance as it reduces the relative risk of a CVD event by 2–4% [39, 40]. In addition, an improvement in insulin sensitivity was reflected by a reduced tAUC, 2-h insulin concentration and the quantitative insulin sensitivity check index. These reductions are clinically meaningful since insulin resistance is a strong predictor of developing T2DM and CVD [41]. Because lipid metabolism and glucose tolerance are important risk factors for the development of cardiometabolic diseases, these multicomponent CWAT-based interventions could be promising for the management and prevention of these chronic diseases.

Multivariate linear regression models from the current study showed a significant association between reduced cardiometabolic risk outcomes and an increase in LPA, independent of the amount of standing time and MVPA. It seems that prolonged sitting was mostly substituted by lower intensity physical activities instead of MVPA, as evidenced by the significant correlation between changes in prolonged sedentary time (bouts > 60 min) and LPA (r = − 0.488, *p* < 0.001; Additional file 1), whereas no correlation between prolonged sedentary time and MVPA was found. This builds on previous experimental studies on the beneficial effects of frequently interrupt sitting time with LPA on cardiometabolic health [27, 35, 42]. This means that it is indicated that individuals who accumulate sedentary bouts of longer duration have a worse cardiometabolic risk profile compared to those with an equal total sedentary time, but regularly interrupt prolonged sitting with LPA. Therefore, it appears that frequently interrupting sedentary behaviour may attenuate the negative effects of sedentary behaviour more than a continuous bout of MVPA. Interestingly, although most experimental studies showed that physical activity interruptions every 20–30 min positively affected cardiometabolic health, our study showed that the same beneficial effects could be achieved with less frequent physical activity interruptions (reduced total time in sedentary bouts of more than 60 min with equal time spent in sedentary bouts < 30 min). Therefore, because LPA is more accessible than MVPA, frequently interrupting sedentary behaviour with LPA could be a promising way for people who fail to reach the recommended levels of MVPA to improve cardiometabolic health and a delayed onset of chronic diseases.

A strength of the study was the use of the ActivPAL™ activity monitor, often referred to as the gold standard for free-living monitoring of sedentary behaviours [43–46], which has the capability to discriminate between postures. In addition, this is one of the few studies that was able to discriminate between the effectiveness of mono- and multicomponent intervention strategies. This study also included participants based on measured time spent in sedentary behaviour instead of physical activity levels, which is often the inclusion criterion in papers within the research field of sedentary behaviour.

However, with respect to the long-term clinical implications, the duration of the interventions period and the extent to which the outcomes are maintained after cessation of the 12-week intervention period should be further investigated. For example, results from meta-analyses have suggested that short-term (< 3 months) sedentary behaviour interventions, as in the current study, have the highest impact, whereas the intervention effects may attenuate after 6 months [12, 47]. Furthermore, although participants were randomly allocated to the control group or one of the intervention groups, we were not able to blind the assessors and participants itself. However, all assessments were performed in an objective way, resulting in less performance bias.

In conclusion, a 12-week multicomponent CWAT-based intervention (CWAT + motivational interviewing) reduces sedentary time, increases physical activity levels and improves various cardiometabolic health variables in sedentary adults. However, self-monitoring in itself (CWAT-only group) has no beneficial effects on sedentary time and additional behaviour change techniques are necessary to effectively reduce sedentary behaviour. From a public health perspective, consumer wearable technology in combination with motivational interviewing may hold the promise for largescale, cost-effective interventions within both healthy individuals and people with cardiometabolic diseases.

## Supplementary Information

**Additional file 1: Figure S1 **Correlations between the difference in sitting time, sitting time of bouts > 60 minutes and physical activity reflected by moderate-to-vigorous physical activity and light intensity physical activity.

## Data Availability

The datasets used and/or analysed during the current study are available from the corresponding author on reasonable request.
