# Supplementary material for: A 12-week consumer wearable activity tracker-based intervention reduces sedentary behaviour and improves cardiometabolic health in free-living sedentary adults: a randomised controlled trial
Source: J Act Sedentary Sleep Behav. 2022 Dec 1;1:8. doi: 10.1186/s44167-022-00007-z (PMC11960220; doi:10.1186/s44167-022-00007-z)
Supplement: Supplementary file 1 — Additional file 1: Figure S1 Correlations between the difference in sitting time, sitting time of bouts > 60 minutes and physical activity reflected by moderate-to-vigorous physical activity and light intensity physical activity. [file 44167_2022_7_MOESM1_ESM.docx]

**Appendix I**

**Figure 1** Correlations between the difference in sitting time, sitting time of bouts > 60 minutes and physical activity reflected by moderate-to-vigorous physical activity and light intensity physical activity.
